# A novel *ex vivo* tumor system identifies Src-mediated invasion and metastasis in mesenchymal tumor cells in non-small cell lung cancer

**DOI:** 10.1038/s41598-019-41301-2

**Published:** 2019-03-20

**Authors:** Aparna Padhye, Christin Ungewiss, Jared J. Fradette, B. Leticia Rodriguez, Jacob L. Albritton, Jordan S. Miller, Don L. Gibbons

**Affiliations:** 1 Department of Thoracic/Head and Neck Medical Oncology, The University of Texas MD Anderson Cancer Center, Houston, TX USA; 2 MD Anderson Cancer Center UTHealth Graduate School of Biomedical Sciences, Houston, TX USA; 3 Department of Molecular and Cellular Oncology, The University of Texas MD Anderson Cancer Center, Houston, TX USA; 4 Department of Bioengineering, Rice University, Houston, Texas USA

## Abstract

Lung cancer is the foremost cause of cancer related deaths in the U.S. It is a heterogeneous disease composed of genetically and phenotypically distinct tumor cells surrounded by heterotypic cells and extracellular matrix dynamically interacting with the tumor cells. Research in lung cancer is often restricted to patient-derived tumor specimens, *in vitro* cell cultures and limited animal models, which fail to capture the cellular or microenvironment heterogeneity of the tumor. Therefore, our knowledge is primarily focused on cancer-cell autonomous aberrations. For a fundamental understanding of lung cancer progression and an exploration of therapeutic options, we focused our efforts to develop an *Ex Vivo* Tumor platform to culture tumors in 3D matrices, which retains tumor cell heterogeneity arising due to *in vivo* selection pressure and environmental influences and recapitulate responses of tumor cells to external manipulations. To establish this model, implanted syngeneic murine tumors from a mutant KRAS/p53 model were harvested to yield multicellular tumor aggregates followed by culture in 3D extracellular matrices. Using this system, we identified Src signaling as an important driver of invasion and metastasis in lung cancer and demonstrate that EVTs are a robust experimental tool bridging the gap between conventional *in vitro* and *in vivo* models.

## Introduction

Lung cancer has the highest mortality rate of all cancer types^1^ primarily because two-thirds of the patients present at a stage when the cancer has already metastasized to distant organs. The morbidity is further exacerbated by a recurrence rate of approximately 50 percent in patients who are treated for early-stage disease and development of resistance to therapeutic agents. Lung tumors display pronounced heterogeneity, including genetically and epigenetically distinct tumor cells surrounded by heterotypic cell types and extracellular matrix that dynamically interact with each of the cell types^2–4^. Experimental cancer research is often restricted to two dimensional *in vitro* cell cultures of immortalized cancer cell lines which largely fail to capture the cellular or microenvironmental heterogeneity of a tumor. For a fundamental understanding of cancer progression and therapeutic vulnerabilities, lung cancer should be studied in a context as close to an *in vivo* setting as possible. However, animal models can be limited by the degree to which conditions can be tested, with added time and expense.

In order to address these deficiencies in current lung cancer models, we established an *Ex Vivo* Tumor (EVT) platform to culture lung tumors in 3D matrices. This system has specific advantages over the more commonly used *in vitro* and *in vivo* systems. First, it retains tumor cell heterogeneity contributed by genetically identical but phenotypically distinct subpopulations arising due to *in vivo* selection pressure and environmental influences^3^. Since the tumors are cultured in a 3D space, the responses of tumor cells to external manipulations like drug treatments are more realistic and can be studied in real time^5,6^. It affords an ability to rapidly test therapeutic sensitivity of tumors in a high throughput manner. Finally, the influences of the tumor microenvironment components can be effectively studied because controlled modifications can be introduced and the system can be tuned to test these interactions^7^. EVTs are intended to bridge the gap between *in vitro* and *in vivo* models for mechanistic and therapeutic study of lung cancer.

Our group and others have previously modeled lung adenocarcinoma using genetically-engineered murine (GEM) systems with mutant KRAS and p53^8^. These GEM models develop lung adenocarcinoma that recapitulates the aggressive and metastatic features observed in patients. Metastasis in this model occurs in a manner that is dependent on an epithelial-mesenchymal transition (EMT) regulated by a double-negative feedback loop between the microRNA-200 family and the ZEB1 transcription repressor^9^. Using syngeneic models derived from these GEMMs, we have previously demonstrated that upon loss of the microRNA-200 family, the mesenchymal tumor cells are dependent on the interaction of the cell adhesion molecule integrin β1 and the extracellular matrix component collagen type I. This interaction drives the formation of the focal adhesion complex through recruitment of the adaptor molecule CRKL, which is a direct miR-200 target^10^. Herein, we make use of the EVT system to investigate the Src signaling pathway downstream of CRKL and demonstrate that lung cancer cells are highly dependent on Src activation for invasion and metastasis. Src is one of the 11 Src-family kinase members, containing an auto-phosphorylation site, Y416, in the activation loop. The tyrosine kinase Src is an oncogene that is overexpressed in many cancer types and known to be involved in multiple cellular processes, such as proliferation, cell morphology, migration, invasion and adhesion^11^. The tyrosine kinase acts as a signal transducer from cell surface receptors (e.g. integrins) through phosphorylation of tyrosine residues on substrates such as FAK, Cas and paxillin^12^.

To establish the EVT model we made use of KP syngeneic murine lung adenocarcinoma tumors^8^, which were isolated, processed and cultured in three-dimensional (3D) matrices. We characterized the behavior of EVTs in different matrices and demonstrate the proof-of-principal for this system to tease out signaling pathways driving metastasis *in vivo*. Our findings demonstrate that matrix-dependent invasion of mesenchymal cells is mediated through activated Src signaling, which is validated by experiments using the *in vivo* models wherein Src inhibition suppresses metastases. Our study also establishes EVTs as a valuable model representative of *in vivo* tumor response. The system presented here can be extended to identify and understand other novel signaling pathways that regulate malignant progression or define therapeutic sensitivities in lung cancer.

## Results

### EVTs are representative of the cellular composition in primary syngeneic murine tumors

**Figure 1:**
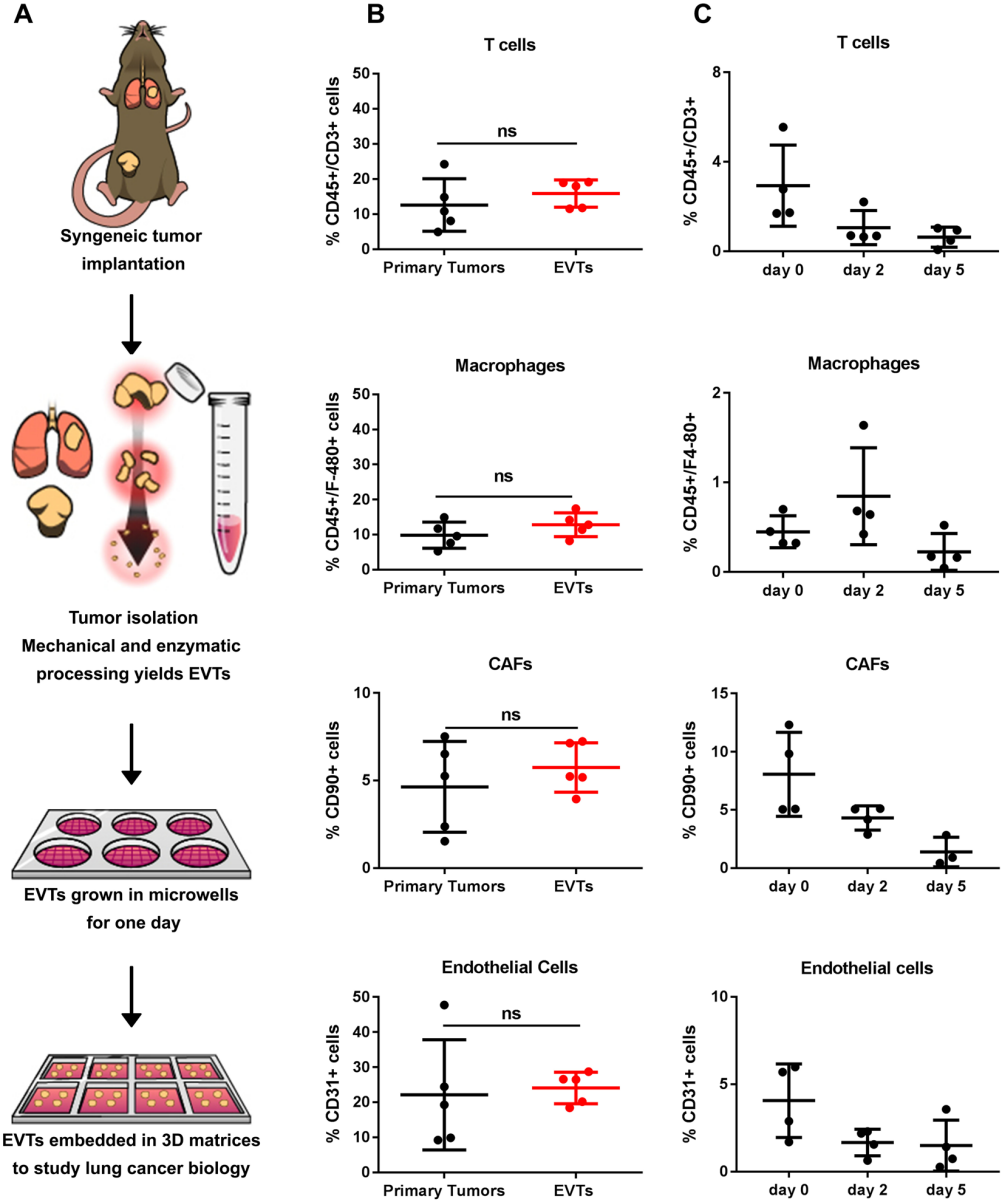
EVTs are representative of the cellular composition of primary syngeneic murine tumors. (**A**) Schematic description of isolation and 3D culture of EVTs from syngeneic murine subcutaneous tumors. Tumors are harvested 4 weeks after implantation. After isolation and processing of tumors, EVTs are cultured in microwells for one day to yield structures of uniform size. (**B**) Flow cytometry analysis to compare the cellular composition of EVTs to the primary syngeneic tumors. No significant difference observed in percentage of T-cells, macrophages, CAFs and endothelial cells between the two groups. (**C**) Flow cytometry analysis to determine the cellular composition of EVTs after culture in Matrigel for 5 days.

To study the underlying mechanisms driving lung cancer progression, we wanted to develop a model that bridges the gap between conventional 2D cell culture systems and *in vivo* models. We utilized murine KP lung adenocarcinoma cell line models (e.g. 344SQ) implanted in immunocompetent syngeneic mice, which form primary tumors that grow over 4–6 weeks and metastasize to the lungs and distant organs^8^. Primary tumors were harvested and processed by mechanical and enzymatic digestion to yield multicellular aggregates, referred to as *Ex Vivo* Tumors (EVTs) (Fig. 1A). Prior to embedding in 3D matrices, EVTs were cultured in laser ablated poly(dimethylsiloxane) (PDMS) microwell inserts designed for standard tissue culture plates, which we have previously described for single-cell suspensions^13^. The microwells within each insert are ~100 µm in diameter, allowing exclusion of tumor aggregates greater than 100 µm and thereby enhancing the uniformity of the starting size for EVTs. EVTs harvested from microwells were then embedded in different matrices for further downstream investigations.

Tumor cells dynamically interact with the components of the tumor microenvironment (TME) like extracellular matrix (ECM), cancer associated fibroblasts (CAFs), immune cells and endothelial cells^4,5^. While establishing this system, we wanted to characterize the cellular TME components present in EVTs that may afford an advantage over conventional 3D cell culture systems. We compared the cellular composition of primary syngeneic tumors and EVTs derived from them by flow cytometry (Fig. 1B). The CD45 cell surface marker was used to identify immune cells within the live cell population, which were further classified as CD3^+^ T-cells or F4/80^+^ macrophages. The two immune cell subpopulations were primarily studied as they have been shown to actively modulate tumor cell behavior. The percentage of T-cells within primary tumors and EVTs was consistent (10–15%) and macrophages followed similar patterns (10–15%). CAFs were identified by the CD90 (Thy1) cell surface marker^14^ and were about 5–7% of the cells in tumors and EVTs. CD31^+^ endothelial cells made up about 20% of the total live cell population in EVTs and primary tumors (Fig. 1B). Figure S1A demonstrates the gating scheme for different TME cells within primary tumor and EVTs. We further analyzed T cell subpopulations further in terms of CD4^+^ and CD8^+^ cells (Fig. S1B). EVTs have a higher percentage of CD8^+^ T cells and lower percentage of CD4^+^ T cells when compared to primary tumors. On further sub-classification into exhausted T cells (PD1^+^/Lag3^+^) and regulatory T cells (CD25^+^/FoxP3^+^), we did not observe any significant differences between tumors and EVTs.

To determine if TME cells were still present in 3D cultured EVTs after a few days without any external stimulation, EVTs were grown in Matrigel for 5 days and the percentage of different cell types was compared over time (Fig. 1C). Although there was a decline in the percentage of TME cells in cultured EVTs without any external stimulation, we were successfully able to detect TME components in EVTs at the time of seeding into matrices and after 5 days of culture, allowing us to mimic an *in vivo* environment. The presence of TME cells within EVTs can be further exploited to investigate the heterotypic interactions within tumors by utilizing appropriate stimulatory factors.

### EVTs are responsive to the external stimulus TGFβ and undergo EMT

**Figure 2:**
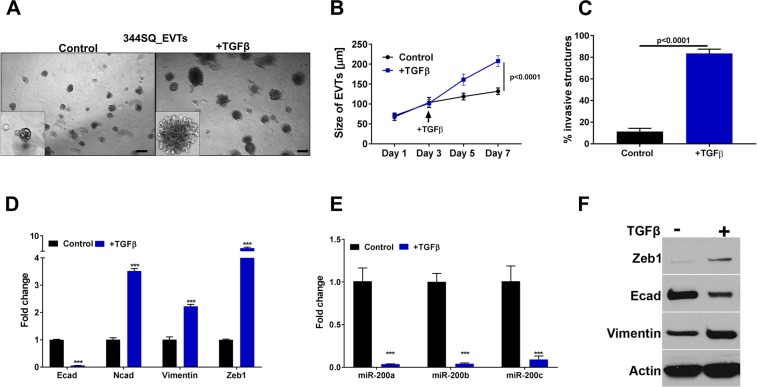
EVTs are responsive to external stimulus TGFβ and undergo EMT. (**A**) 344SQ_EVTs are cultured in Matrigel for 7 days and treated with TGFβ (5 ng/ml) on day 3 (scale bar is 200 µM). In response to TGFβ, EVTs (**B**) hyper proliferate and (**C**) are more invasive. For each condition, 1000 structures were measured in size and scored for invasiveness. (**D**) Quantitative RT–PCR of indicated markers, shown as fold change upon TGFβ treatment. (**E**) Taqman RT–PCR assay of individual miR-200 members for EVTs in Matrigel and following TGFβ treatment. ***p < 0.0001. (**F**) Western blot analysis for EMT markers in Matrigel with or without TGFβ treatment.

Tumors are surrounded by extracellular matrix, which provides physical support as well as biochemical signals to tumor cells, and is frequently altered over the course of tumor progression. We initially utilized Matrigel, which is a solubilized basement membrane preparation rich in primarily laminin and collagen IV, to model the ECM-EVT interactions found in tumors. 344SQ_EVTs were cultured in Matrigel for 7 days and treated with TGFβ starting on the third day after seeding. TGFβ is frequently present at high levels in tumors and is known to induce an EMT by suppression of miR-200 expression^8^, thereby promoting invasion and metastasis. EVTs grown in Matrigel alone increase from ~75 µm to ~100 µm in size over 7 days, but upon treatment with TGFβ, they hyper-proliferate and are ~200 µm by day 7 (Fig. 2A,B). TGFβ treated EVTs developed invasive protrusions suggesting a shift to a mesenchymal phenotype (Fig. 2A,C). To confirm that EMT is occurring at a molecular level, we collected RNA and protein from EVTs at day 7. Quantitative RT-PCR analysis for canonical EMT markers E-cadherin, N-cadherin, vimentin and Zeb1 revealed that EVTs undergo EMT upon TGFβ treatment (Fig. 2D,E). Consistent with our previous work using murine lung cancer cells alone^8^, with EVTs we observed an increase in Zeb1 in response to TGFβ, with subsequent transcriptional repression of the miR-200 family members miR-200 a, b, c (Fig. 2E). Additionally, we observed an increased expression of mesenchymal markers at the protein level by western blot analysis (Fig. 2F).

To demonstrate the broader utility of the system, we intratracheally implanted RFP expressing 344SQ tumor cells into the lungs and harvested orthotopic lung tumors after three weeks of growth. H&E staining of lungs reveal presence of tumors formed from implantation of tumor cells. Lung-EVTs were cultured in Matrigel over 7 days and demonstrated similar morphological and molecular characteristics as EVTs derived from subcutaneous tumors (Fig. S2A). In response to TGFβ, they developed an invasive morphology corresponding to the changes in EMT markers at the molecular level (Fig. S2B).

### Extracellular matrix manipulations induce phenotypic alterations in EVTs

**Figure 3:**
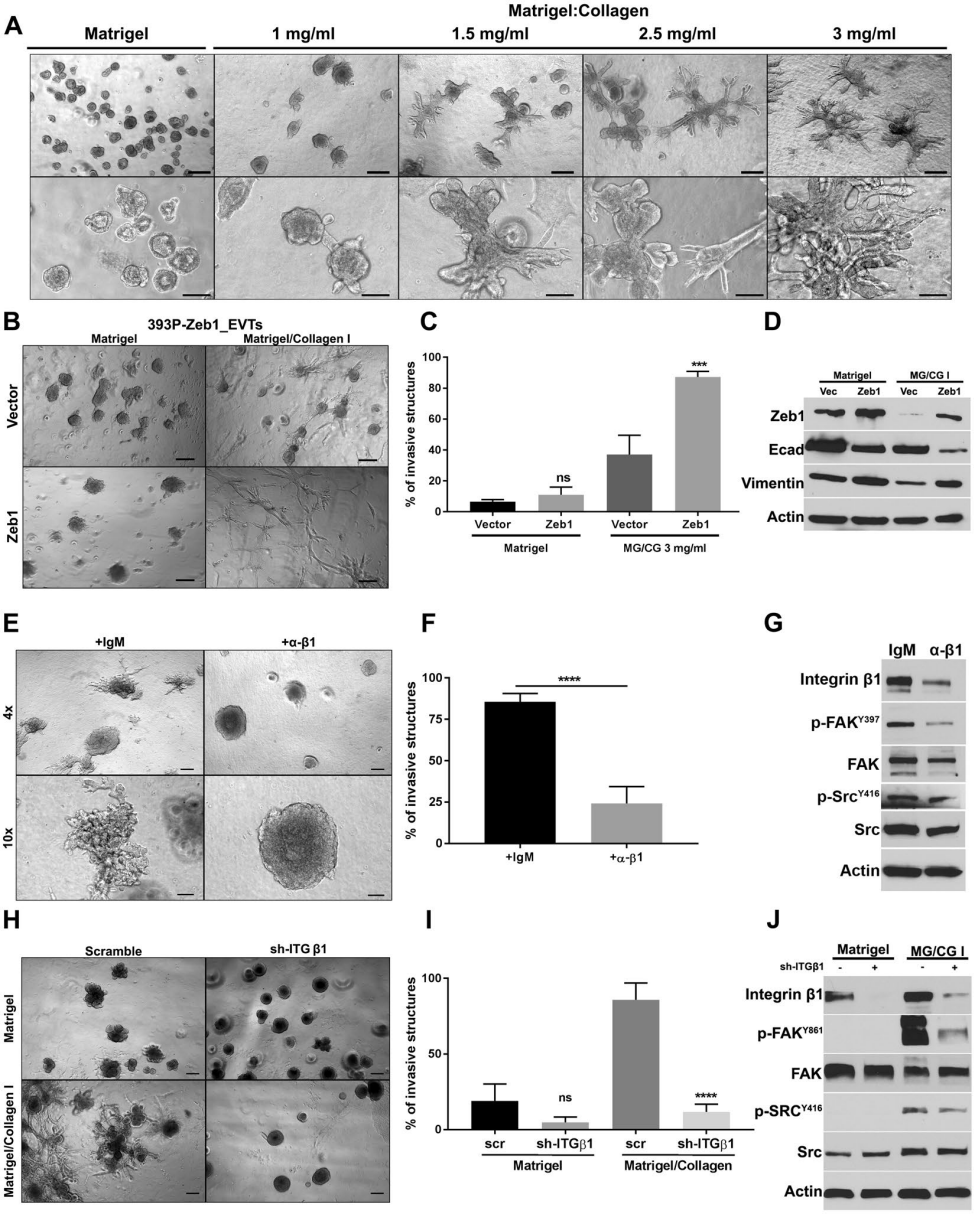
Extracellular matrix manipulations induce phenotypic alterations in EVTs. (**A**) EVTs are cultured in Matrigel or Matrigel/Collagen I with different concentrations of Collagen I for 5 days. (**B**) 393P_vector and 393P_Zeb1 EVTs are cultured in Matrigel and Matrigel/Collagen I (3 mg/ml) for 5 days (scale bar is 200 µM). (**C**) Invasiveness of EVTs in response to intrinsic (Zeb1) or extrinsic (Collagen I) manipulation is compared. Percentage of invasive structures were calculated for each condition. ***p < 0.0001. (**D**) Western blot analysis to show an EMT is induced with Zeb1 overexpression but not with matrix alteration. (**E**) 344SQ_EVTs are cultured in Matrigel/Collagen I (3 mg/ml) and treated with an ITGβ1-blocking antibody or IgM control for 5 days. (**F**) For each condition, 500 structures were scored for invasiveness. (**G**) Western blot analysis of FAK/Src signaling in EVTs treated with ITGβ1-blocking antibody. (**H**) 344SQ_scr and 344SQ_shITGβ1 EVTs are cultured in Matrigel and Matrigel/Collagen I (3 mg/ml). Images taken at day 5 and scale bar is 200 µM. (**I**) Percentage of invasive structures were calculated for each condition. ***p < 0.0001 (**J**) Western blot analysis to show integrin β1 knockdown and FAK/Src signaling.

One of the factors contributing to intratumor heterogeneity is the plasticity of tumor cells to transition between different phenotypic states. Tumor cell intrinsic genetic or epigenetic alterations are usually considered drivers of cancer progression. However, modification of the extracellular matrix can independently modulate the phenotype of tumor cells with or without affecting the epigenetic state of the tumor cells. To test the effects of matrix composition on tumor behavior, we cultured 344SQ_EVTs in Matrigel or mixed in increasing concentrations of collagen I. The presence of higher amounts of collagen I has been shown to promote an invasive and more aggressive tumor phenotype. We observed that with each higher collagen I concentration the percentage of invasive EVTs significantly increased up to ~80% (Figs 3A and S3A). These data demonstrate that ECM components are capable of driving the phenotypic behavior of the tumor cells. To determine if interaction with collagen I is altering the tumor cells by inducing an EMT, we collected RNA and protein from the structures. Quantitative RT-PCR (Fig. S3B) and western blot analysis (Fig. S3C) did not reveal an EMT occurring in EVTs, demonstrating that ECM is driving the invasive phenotype of the tumor cells without inducing epigenetic changes that result in a mesenchymal state.

To test the utility of this platform across different tumor cell types, we harvested EVTs from non-metastatic, epithelial 393 P tumors. 393 P_EVTs cultured in Matrigel grew as aggregates which were significantly invasive in response to TGFβ treatment or inclusion of collagen I in the matrix (Fig. S3D). An invasive phenotype was also observed with EVTs from orthotopic lung tumors cultured in Matrigel/Collagen I (Fig. S3E). Our previous work has revealed that Zeb1 expression drives a mesenchymal phenotype in non-metastatic, epithelial cell line 393 P^10^. EVTs derived from genetically manipulated 393 P cells with constitutive Zeb1 expression were cultured in Matrigel or Matrigel/Collagen I and displayed significant invasion in MG/CG I as compared to the 393P-Vector_EVTs (Fig. 3B–D). Overall, testing of multiple different tumor cell models demonstrates the utility of the EVT platform to study the dynamic interactions of cell intrinsic factors with the extracellular conditions.

To further study the mechanism by which collagen I promotes an invasive phenotype, we cultured 344SQ_EVTs in Matrigel/Collagen I at 3 mg/ml concentration and treated with an integrin β1 blocking antibody. The antibody decreased the invasive ability of EVTs by two-thirds (Fig. 3E,F). Western blot analysis also demonstrated that FAK/Src signaling is activated in the presence of collagen I and is suppressed by addition of integrin β1 blocking antibody (Fig. 3G). We tested the effect of integrin β1 blocking antibody on EVTs in Matrigel alone (Fig. S3F). In the absence or presence of the blocking antibody, we did not observe any invasion of EVTs in Matrigel, further demonstrating that integrin β1 receptors on tumor cells require interaction with collagen I in the matrix to produce invasion.

To further test that invasion in EVTs is occurring through an integrin β1-collagen I interaction, we cultured EVTs of 344SQ with integrin β1 knockdown (344SQ_ITGB1 KD) in Matrigel and Matrigel/collagen I (Fig. 3H). There was a significant decrease in the percentage of invasive EVTs in Matrigel/Collagen I upon integrin β1 knockdown as compared to EVTs from scramble control cells (Fig. 3I). Western blot analysis showed a decrease in p-FAK and p-Src in integrin β1 knockdown EVTs (Fig. 3J). These results demonstrate the utility of EVTs for interrogating the role of TME components like ECM in cancer progression and highlight the collagen I-integrin β1 interactions as critical to downstream signaling and tumor cell invasion.

### Src pathway is activated in mesenchymal cells and required for initiation and maintenance of invasion in EVTs

**Figure 4:**
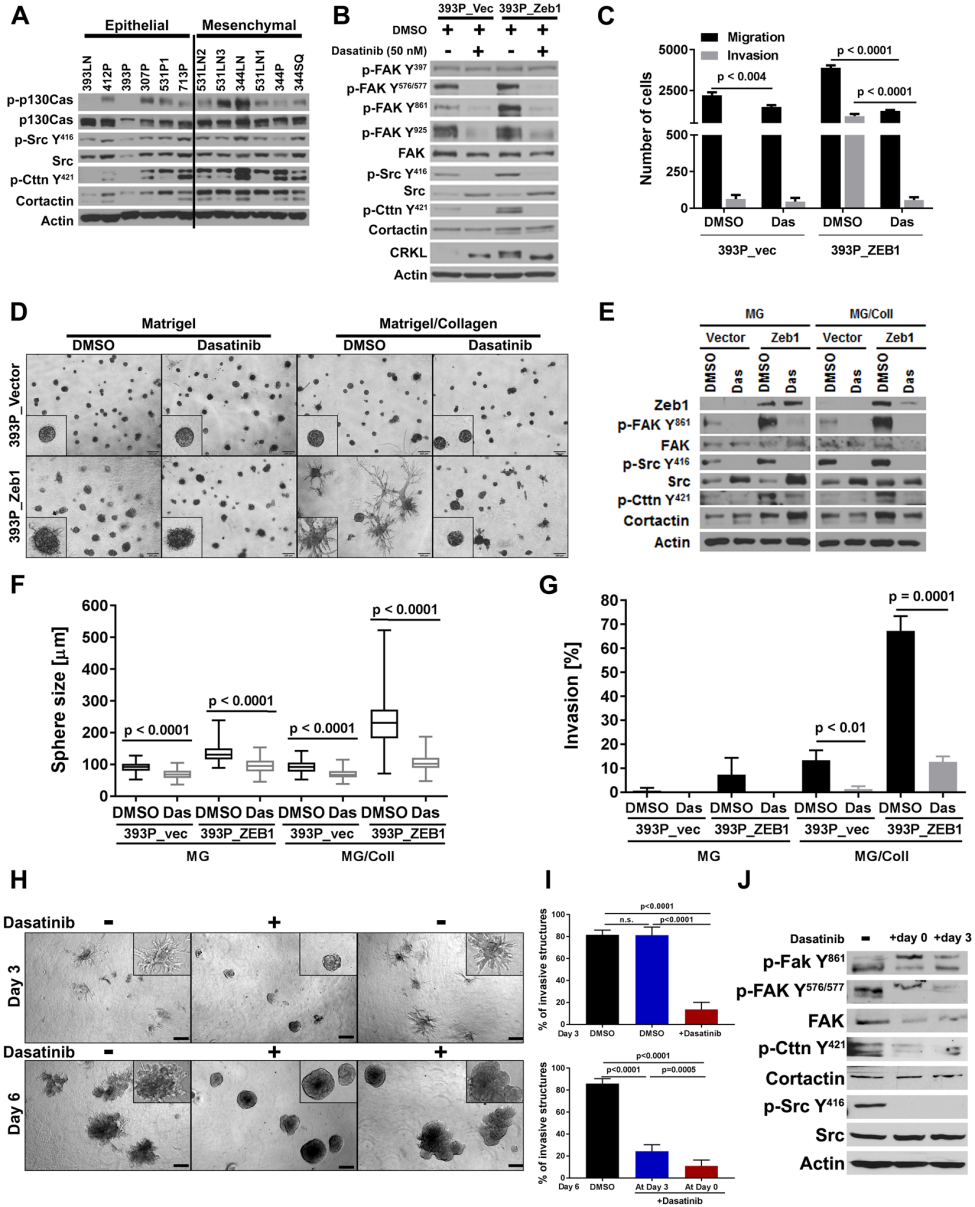
Src pathway is activated in mesenchymal cells and required for initiation and maintenance of invasion. (**A**) Western Blot analysis shows increased Src pathway activation in the mesenchymal cells lines and in cell lines stably expressing Zeb1 (**B**), which is inhibited upon a 7 hr treatment with dasatinib. (**C**) Dasatinib treatment inhibits the migratory and invasive ability of mesenchymal cells in Transwell assays. (**D**) 393P_Zeb1 and control cells grown in 3D cultures. Invasion of 393P_Zeb1 cells in a mixture of Matrigel/Collagen I (1.75 mg/ml) is inhibited upon dasatinib treatment (50 nM). Images were taken at day 7 after a 3 day treatment. (**E**) Western blot analysis of dasatinib treatment in 3D assays in Matrigel and Matrigel/Collagen I shows inhibition of the Src pathway. Quantification of (**F**) sphere size and (**G**) invasiveness of cells in 3D cultures from (**D**). Represented is the average of 3 wells, each measuring 30 structures in size and scoring 50 structures for invasiveness. (**H**) Dasatinib treatment of EVTs cultured in Matrigel/Collagen I (3 mg/ml) before (at day 0) and after initiation of invasion (day 4). Images were taken at day 3 and day 6. (**I**) Quantification of invasive structures before and after dasatinib treatment. For each condition, 500 structures were scored for invasiveness. (**J**) Western blot analysis shows Src-signaling is inhibited.

We previously demonstrated that the EMT status of tumor cells modulates collagen I-dependent activation by regulating integrin β1-mediated FAK activation^10^. We sought to further investigate the signaling downstream of integrin β1 that accounts for the invasive phenotype. Stratification of murine KP cell lines derived from spontaneous Kras/p53 tumors by their epithelial or mesenchymal status (Fig. S4A) revealed a significant activation of the downstream Src/cortactin pathway in the mesenchymal cells compared to the epithelial cells (Fig. 4A). Furthermore, in the genetically manipulated 393 P cells, we observed a direct correlation between the activation of this pathway and the Zeb1 levels (Figs 4B and S4B). To further study the importance of the Src pathway in regulating invasion, we assayed the effect of the Src tyrosine kinase inhibitor, dasatinib. Dasatinib treatment for 7 hours blocked FAK and Src activation (Fig. 4B) and significantly reduced 2D migration and invasion, suppressing the highly invasive 393P_ZEB1 cells to the baseline control levels (Fig. 4C). Dasatinib treatment in 3D assays inhibited the collagen I-dependent invasion of the mesenchymal 393P_ZEB1 cells but had no effect on the control cells (Fig. 4D–G). Dasatinib treatment also inhibited migratory and invasive ability of the human lung cancer cell line H157 in Boyden chamber assays and caused a single cell or tiny cluster phenotype in 3D assays (Fig. S4C–E).

To further elucidate the utility of EVTs for investigating signaling pathways responsible for lung cancer invasion and metastasis, we cultured EVTs derived from 344SQ tumors in Matrigel. This is one of the KP models that is mesenchymal and metastatic, owing to its ability to undergo epigenetic changes and readily respond to the external microenvironment, including ECM changes. 344SQ_ EVTs grown in Matrigel/Collagen I were tested for their dependency on Src signaling for invasion. Three groups were setup, with one serving as control without dasatinib, another received dasatinib at the time of seeding and the third group was allowed to invade in collagen I for 3 days before dasatinib was added to the culture. Dasatinib was able to significantly suppress invasion in both early and late conditions, emphasizing that Src is necessary for initiation and maintenance of invasion (Fig. 4H,I). Western blot further confirmed that the Src pathway is inhibited with dasatinib treatment (Fig. 4J).

### Migration, invasion & TGFβ response of cells and EVTs are blocked with Src inhibitors

**Figure 5:**
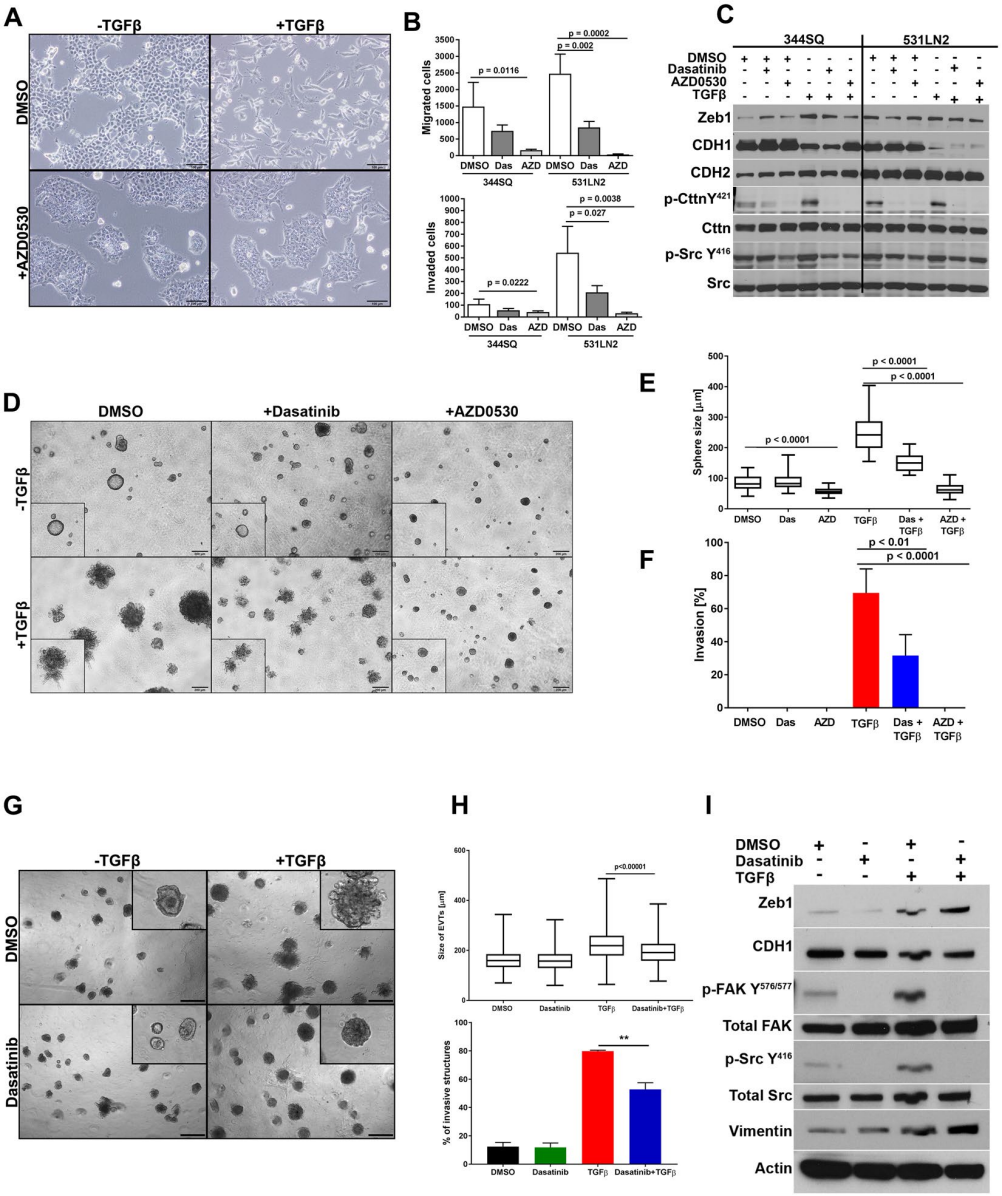
Migration, invasion & TGFβ response of cells and EVTs are blocked with Src inhibitors. (**A**) Morphology of the 344SQ cells in the indicated treatment conditions. (**B**) The migratory and invasive Transwell assays upon DMSO, Dasatinib (50 nM) or AZD0530 (3 μM) treatment. (**C**) Western Blot analysis of 344SQ and 531LN2 cells treated with Src inhibitors and TGFβ at the indicated concentrations for 3 days. (**D**) Sphere formation of 344SQ in 3D Matrigel cultures. Images were taken at day 10 and sphere size (**E**) and invasiveness scored (**F**). Dasatinib (50 nM) was added at day 4, AZD0530 (3 μM) at day 5 and TGFβ (5 ng/ml) at day 6. (**G**) EVTs culture in Matrigel for 8 days. Images were taken at day 8. Dasatinib (50 nM) was added at the time of seeding and TGFβ (5 ng/ml) at day 4. (**H**) Size of EVTs and invasiveness of EVTs on TGFβ and dasatinib treatments. (**I**) Western blot analysis of EVTs treated with Dasatinib and TGFβ at indicate concentrations.

TGFβ is a well-known EMT inducer to which we have previously shown the metastasis-prone KP cells are quite sensitive. We next tested if Src inhibition would block the TGFβ-induced EMT. We utilized dasatinib and a second Src inhibitor, AZD0530 (saracatinib). 344SQ cells treated with TGFβ alone showed a fibroblastic phenotype, whereas cells that received TGFβ and AZD0530 displayed an epithelial morphology (Fig. 5A). Similar to the results with the invasive 393P_ZEB1 cells, treatment with either Src inhibitor decreased Transwell migration and invasion of the mesenchymal 344SQ and 531LN2 cells (Fig. 5B). Dasatinib and AZD0530 blocked Src pathway activation even in combination treatment with TGFβ and showed a decrease of Zeb1 (Fig. 5C). In 3D culture of isolated cancer cells, treatment of 344SQ cells with TGFβ caused the spheres to hyper-proliferate and become highly invasive (Fig. 5D). We previously showed that inhibition of integrin β1 or the adaptor molecule CRKL significantly decreased the ability of the cells in 3D cultures to become invasive upon TGFβ treatment^10^. A similar result was seen in the combined treatment of TGFβ with the Src inhibitors, leading to a significant reduction in sphere size and invasion, which was more pronounced with AZD0530 treatment (Fig. 5E,F).

To assess the dependency of EVTs on Src signaling for invasion *in vitro* and *ex vivo*, we cultured 344SQ_EVTs in Matrigel, which were treated with Src inhibitors alone or in combination with TGFβ. EVTs hyper-proliferated and developed invasive protrusions in response to TGFβ. This phenotype was suppressed in EVTs pre-treated with dasatinib (Fig. 5G,H) or AZD0530 (Fig. S5). TGFβ caused an increase of canonical mesenchymal markers like Zeb1 and Vimentin and suppressed E-cadherin levels. There was also an upregulation of p-FAK and p-Src in response to TGFβ. However, Src inhibition was able to overcome TGFβ mediated FAK/Src pathway activation and the subsequent invasive phenotype, despite the TGFβ-induced EMT in EVTs observed by western blot (Fig. 5I). These results illustrate that the Src pathway is activated through integrin β1 in an ECM-dependent manner and cell invasion can be inhibited by targeting the cell:ECM interactions or signal transduction pathways *in vitro* and *ex vivo*. In addition, we were able to demonstrate the strength of EVTs to facilitate the interrogation of tumor phenotype at multiple levels, in a high throughput manner that would not be otherwise easily attainable *in vivo*.

### Dasatinib treatment *in vivo* decreases lung and distant organ metastases

**Figure 6:**
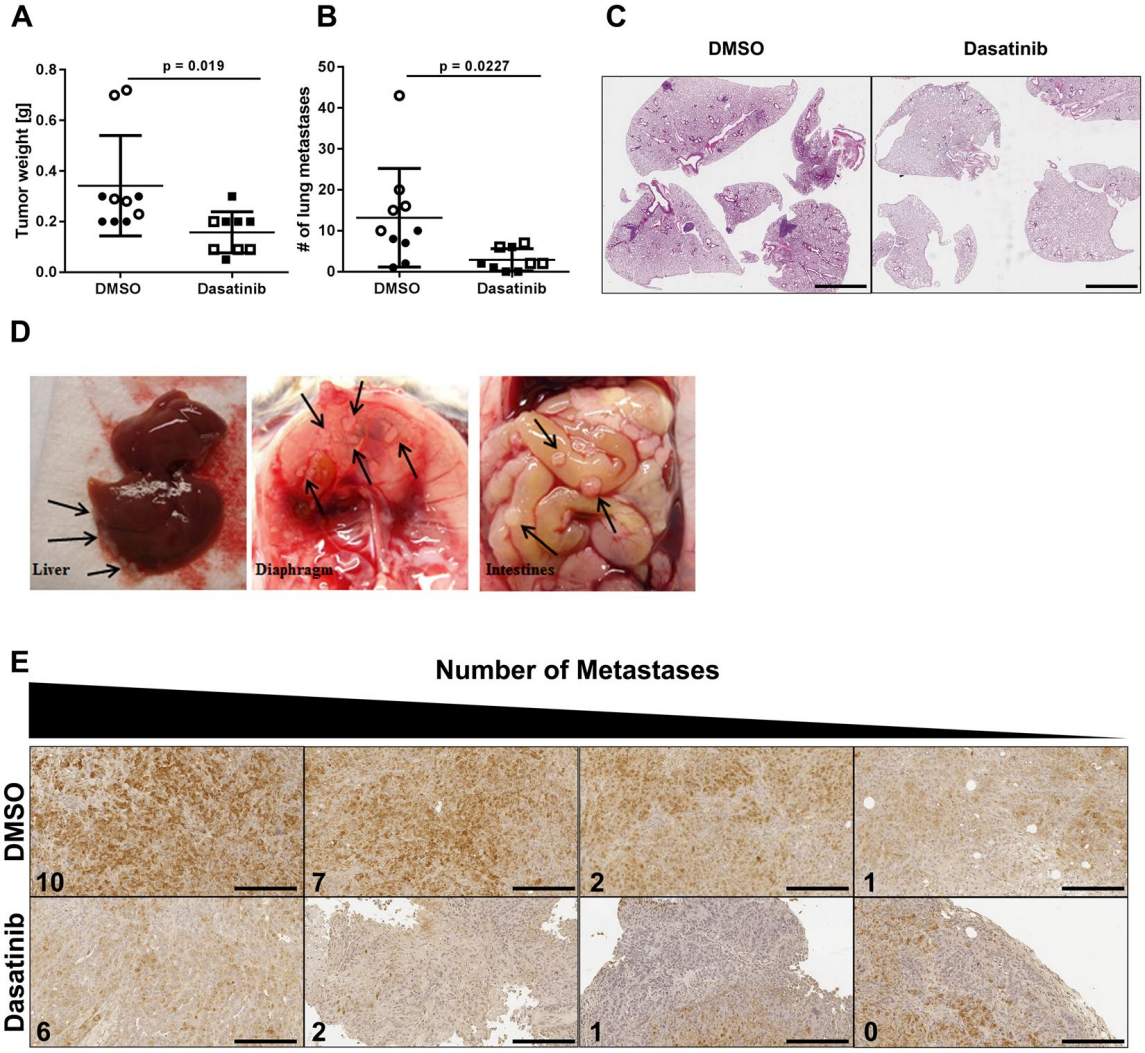
*In vivo* dasatinib treatment decreases lung and distant organ metastases. (**A**) Primary tumor weight and (**B**) number of lung metastases in syngeneic mice treated with Dasatinib, n = 9 (10 mg/kg: solid shapes, 20 mg/kg: empty shapes) or vehicle, n = 10. (**C**) H&E staining of the lung sections with and without Dasatinib treatment. (**D**) Images of sites of distant metastases from DMSO treated animals (black arrows indicate metastases). (**E**) Immunohistochemistry of intratumoral p-Src levels in the DMSO and Dasatinib treated mice.

The 344SQ cells are paradigmatic of the highly metastatic KP cells in our syngeneic murine model^10^. To assess whether the EVT results were predictive of the *in vivo* activity of dasatinib to block the Src signaling pathway and suppress metastases, we injected syngeneic mice with 344SQ cells and dosed them with 10 or 20 mg/kg Dasatinib 5 days/week. The results show that treatment at either concentration significantly reduced the number of lung metastases (Fig. 6A,B), as shown in the H&E-stained lung sections of mice treated with DMSO or with dasatinib (Fig. 6C). Moreover, metastases were found in the liver, kidneys, spleen, intestines and diaphragm in the control mice but were not observed in the dasatinib-treated mice (Fig. 6D). Immunohistochemical staining of the primary tumors demonstrated a strong correlation between p-Src levels and the number of distant metastases (Fig. 6E), with the highest number of metastases found in mice with the highest intratumoral p-Src staining.

## Discussion

The role of intra- and inter-tumor heterogeneity in driving tumor metastasis and therapy resistance has been greatly emphasized in recent years^5,15^. Tumors are not just a mass of cancer cells, but instead are composed of a heterogeneous tumor microenvironment, including changes in the ECM and heterotypic cell types, that dynamically interacts with tumor cells. The diverse selection pressures from the microenvironment profoundly affect the behavior and phenotype of cancer cells and must be taken into account while studying mechanisms that drive cancer progression. The goals of this study were to establish a unique system to better model *in vivo* conditions, identify mechanisms driving invasion of mesenchymal lung cancer cells and demonstrate the ability to identify therapeutic sensitivities of tumor cell subsets dependent upon the microenvironmental interactions.

The *Ex Vivo* Tumors (EVTs) utilized in this study are micro-tumors derived from immunocompetent murine syngeneic tumors and cultured in 3D matrices. We determined that EVTs faithfully represent the cellular composition of tumors and retain the heterotypic heterogeneity within tumors. A decline of heterotypic cell types was observed in culture over time in the absence of additional stimulatory factors. However, such factors can be included in subsequent studies in order to study specific interactions of tumor cells with heterotypic cell types. These features demonstrate that the EVT platform can be used to study micro-tumors and investigate influences of non-tumor cells in a high throughput manner which cannot be achieved with conventional 2D or 3D cell cultures.

EVTs were functionally characterized by investigating their responses to external manipulations like growth factors and matrix alterations. Using a laminin rich matrix we recapitulated a basement membrane-like microenvironment and EVTs were non-invasive even though primary tumors are highly metastatic^8^. This underlines the importance of architecture and contextual cues in growth of tumor cells which are lost in monolayer cell cultures. TGFβ induced a central EMT and invasive phenotype in tumor cells. ECM modification promoted an invasive phenotype in EVTs without altering the EMT state of the tumor cells. This finding is in contrast to previously observed collagen I-mediated EMT in tumor cells through autocrine TGFβ signaling^16^. However, it is known that ECM components actively interact with tumor cells for malignant progression through a variety of signaling cues modulating cellular behavior, ranging from tumor cell survival to invasion and metastasis^17,18^ and TGFβ-mediated signaling may be independent of collagen I influence. It also indicates that EVTs are more amenable to dynamic phenotypic alterations, which may be attributable to the presence of distinct tumor cell subpopulations. This emphasizes the importance of investigating TME-tumor cell interactions in the context of tumor cell heterogeneity. Monolayer cell cultures of tumor cells are unable to capture this heterogeneity and hence have limited biological relevance.

Based on the effect of ECM composition on tumor cell phenotype observed in our system, we investigated the signaling downstream of collagen I-integrin β1 interactions as responsible for driving invasion and metastasis. An increased activation of Src signaling pathway molecules in mesenchymal cells suggested a dependency on p-Src for their invasive phenotype. Src inhibition has been shown to decrease migration *in vitro*, tumor growth and invasion *in vivo* in NSCLC using murine xenograft models^19,20^. Furthermore, inhibition of Src using the ATP binding competitive inhibitor dasatinib decreased the development of liver metastases in a murine model of pancreatic carcinoma and caused a decrease in cell adhesion, migration and invasion in colon cancer cell lines. In thyroid cancer cell lines, dasatinib was shown to have a cytostatic activity both *in vitro* and *in vivo* causing cell cycle arrest and an increase in senescence^21^. Tumor cells in our 3D matrix experiments demonstrated that Src-mediated invasion was substrate dependent. Type I collagen in the matrix enhanced p-Src expression in tumor cells, which was abrogated upon treatment with Src inhibitors. Pharmacological inhibition of Src was sufficient to prevent initiation and maintenance of invasive structures in 3D cultures with collagen I, further demonstrating the importance of Src signaling in lung cancer cells. TGFβ is a well-known inducer of EMT and subsequent invasive phenotype. TGFβ has also been shown to mediate cell adhesion to ECM and increased collagen I synthesis in a Src-dependent manner^22^. We wanted to test the hypothesis if Src inhibition is sufficient to overcome TGFβ-mediated invasion. A combination treatment with TGFβ and pharmacological Src inhibitors prevented invasion, despite EMT, emphasizing the high dependency of tumor cells on Src signaling. These findings were in concert with another study which demonstrated Src inhibition could overcome TGFβ induced myofibroblast differentiation^23^. Furthermore, *in vivo* studies showed a significant decrease in metastases with Src inhibition. We identified collagen I-integrin β1 activated Src signaling as a major driver of invasion and metastasis in lung cancer. Combined with our previous findings, integrin β1 and Src are interesting targets for a combinatorial treatment approach in lung cancer.

Invasion mediated through the Src signaling pathway demonstrates the utility and applicability of EVTs as a potent tool for mechanistic interrogation of invasion and metastasis in lung cancer. The tumor model presented here incorporates tumor cell and tumor microenvironment heterogeneity arising *in vivo* in response to different selection pressures. It also retains the ability of tumor cells to respond to the intrinsic (Zeb1 expression in cells) and extrinsic manipulations (matrix and soluble factors). This suggests that phenotypic differences between tumor cells arise due to their differing ability to interact with the stromal components around them as much as due to genetic differences.

Our next step is to expand the EVT system by introducing cellular complexities and accounting for the effect of tumor cell and tumor microenvironment heterogeneity. Early work led by Bissell and colleagues in the field was landmark to establish the significance of three-dimensional models in normal breast epithelial differentiation and tumor cell morphogenesis^24–27^. The role of cell-cell and cell-matrix interaction has been widely emphasized for recapitulating tissue architecture, tissue morphogenesis, and epithelial organization and these aspects become significantly more important when investigating biological phenomenon in cancer cells. Following the pioneering work in normal and malignant breast tissue, the field rapidly expanded to simulate 3D growth of different organs such as skin^28^, bone^29^, brain^30^, pancreas^31^ and prostrate^32^. Currently, the most common three dimensional systems to recapitulate tumor growth are usually monocultures of cancer cells^6^ or co-cultures to study heterotypic interactions between tumor cells and other cell types like CAFs or immune cells^7,33^ where multicellular spheroids are cultured in artificially created environments. A common objective for many of these studies is to serve as an intermediate between the use of whole animals at one end of the spectrum and cellular monolayers at the other, but are limited to studying a single TME component or a unidirectional effect. EVTs have a distinct advantage of recruiting TME cellular components and retaining the integrity of a tumor which confers the ability to investigate specific roles of TME components in the tumor progression^34^. In addition to heterotypic heterogeneity, we are able to account for tumor cell heterogeneity in the system. We have a large panel of murine NSCLC cell lines isolated from Kras/p53 GEM model from the primary and metastatic tumor sites^8^, which demonstrate phenotypic differences. When re-implanted subcutaneously or orthotopically in syngeneic animals, the cell lines demonstrate varying metastatic potential and differential interactions with the TME components *in vitro*^10,35–37^. Therefore, EVTs derived from different cell types can allow us to tease out the tumor cell heterogeneity modulated by cell intrinsic and extrinsic manipulations, creating a robust tool for investigating lung cancer progression.

Multiple groups have also demonstrated that in addition to recapitulating tissue morphology, the intracellular signaling characteristics are more accurately represented in 3D cultures^38–41^. Birgersdotter *et al*.^42^, Li *et al*.^43^ and Luca *et al*.^44^ demonstrate considerable differences in gene expression and mRNA splicing patterns when cells are cultured under 2D versus 3D conditions. Other studies have utilized 3D tumor spheroid-based functional assays for target validation and drug evaluation and have demonstrated that sensitivity and response of cancer cells to different compounds can vary between 2D and 3D cell cultures^45^. Organization of tumor cells in polarized structures in an integrin β4-dependent manner imparted resistance to apoptosis in mammary epithelium suggesting signaling pathways determining sensitivity to drugs can vary based on the tissue architecture^46^.

It is now widely accepted that the response of tumor cells to drug treatments is not dependent on oncogenic drivers alone. The TME plays a very significant role in modulating the outcome of therapeutic interventions^5,47–51^. Multiple groups have attempted to capture the complexity of the microenvironment and test therapeutic sensitivity to drug treatments. For example, tumor cells have been cultured in different matrices to identify ECM combinations driving lung cancer^49,52^. Work done by such groups has given great insights into individual heterotypic interactions, however, these studies have been conducted using cell lines in an artificially created environment. Our tumor model incorporates cells from a tumor’s microenvironment allowing us to maintain the complexity of the tumors in a physiologically relevant context. Building on the groundwork in the present study, this approach can be utilized in a high throughput manner to investigate tumor cell and microenvironment heterogeneity in lung cancer metastasis and resistance to drug treatments.

## Materials and Methods

### Murine lung cancer cell lines and syngeneic mouse model

All animal experiments were reviewed and approved by the Institutional Animal Care and Use Committee at The University of Texas M.D. Anderson Cancer Center performed in accordance to their guidelines. Primary and metastatic murine lung cancer cell lines previously derived from spontaneous Kras/p53 GEMMs^8^ were cultured in RPMI 1640 with 10% FBS. 344SQ cell lines are metastatic *in vivo* upon subcutaneous implantation. Wild-type 129/SV mice from our colony (males and females) of at least 8 week of age were used for the syngeneic tumor experiments. Subcutaneous injections of 1 million cells in single-cell suspension were placed in the posterior flank in 100 µL of media. Animals were monitored regularly and euthanized when they exhibited signs of morbidity or when size of the subcutaneous tumor required sacrifice (5–6 weeks). For intratracheal implantation of tumor cells, mice were anesthetized via intra-peritoneal injection of room temperature 20 mg/ml avertin and intubated as previously described^53^. 2.5 × 10^4^ cells in single-cell suspension of 50 µL of media were delivered through trachea and were allowed to grow for three weeks before harvesting. The animals received intraperitoneal injections 5 days/week with DMSO or dasatinib (purchased from Selleckchem) at a dose of 10 or 20 mg/kg in 50 μl. Mice were examined for metastasis and tissues for the subcutaneous tumor, lungs and any organs with visible metastasis were collected. The results are represented as mean ± standard deviation and student’s t-test was performed for statistical significance.

### *Ex Vivo* Tumors: isolation and processing

Primary subcutaneous and orthotopic lung tumors were isolated and collected in PBS-10% FBS solution at 6 weeks after implantation. Tumor tissues were initially subjected to mechanical breakdown by mincing the tumors with scalpel followed by chemical digestion in 10 ml of 0.2% Collagenase A (Sigma Aldrich 10103578001), 0.2% trypsin (Gibco 27250018), 0.5% FBS in RPMI for one hour at 37 °C. Tissue suspension was further broken down in a gentleMACS™ Dissociator using the lowest setting to yield multicellular tumor aggregates. The solution was centrifuged at 1500 rpm for 10 min to remove Collagenase/trypsin from the solution and incubated with DNAase (2 U/µL) for 5 minutes at room temperature. Three rounds of differential centrifugation were performed to remove any single cell from the EVTs. EVTs were cryopreserved in 90% FBS-10% DMSO solution for future use.

### 3D Culture

The processed EVTs were cultured for one day in poly(dimethylsiloxane) (PDMS) microwell inserts designed for standard tissue culture plates^13^. The microwells within each insert are ~100 µm in diameter allowing exclusion of aggregates greater than 100 µm and yielding EVTs of uniform size. EVTs harvested from microwells were embedded in 100 µL of Matrigel (BD 356231), plated on 8-well chamber slides pre-coated with 25 µL of Matrigel and incubated at 37 °C for 30 minutes. The cultures were supplemented with media containing RPMI 1640, 10% FBS, 2% Matrigel and 1% Penicillin-Streptomycin.

### Invasion assay

Invasive potential of EVTs was tested in response to Transforming Growth Factor β (TGFβ) (CST #8915LC) in the media and rat tail Collagen I (BD 354249) in the matrix. TGFβ at 5 ng/ml was added to the media and replenished every 48 hrs. EVTs were scored as invasion-positive in response to TGFβ if one or more protrusive structures were present. EVTs were embedded in Matrigel/Collagen mixture, plated on 8-well chamber slides pre-coated with 25 µL of Collagen mix at the indicated concentration, incubated at 37 °C for 45 minutes to allow polymerization of Collagen and then supplemented with RPMI 1640 containing 10% FBS, 2% Matrigel and 1% Penicillin-Streptomycin. The structure sizes and invasion were scored at the end of the experiment, with structures counted invasion-positive in Matrigel/Collagen mixture if one or more protrusions were present.

### Flow cytometry on EVTs for TME components

For isolation of single cells from EVTs, media was removed from the wells and washed with PBS. EVTs were collected in 50 mL falcon tubes with 10 mL of cold PBS-EDTA and shaken at 4 °C for 30 minutes. The solution was centrifuged, supernatant removed and resuspended in Collagenase-trypsin mix. This was shaken at 37 °C for 30 minutes followed by neutralization with complete media. Supernatant was removed after centrifugation and cells were resuspended in 100 µL of FACS buffer (PBS + 5% FBS). Samples were stained with conjugated flow cytometry antibodies for TME components. Zombie Aqua (Biolegend 423101) was used as a marker to detect live cells. CAFs were detected with CD90.1 antibody (PerCP Mouse Anti-Rat CD90/Mouse Catalog No. 557266), endothelial cells were detected with CD31 antibody (PE anti-mouse Catalog No. 102407) and immune cells were detected with CD45 antibody (FITC anti-mouse Catalog No. 103108). To further identify immune cells, we used CD3 antibody (PE/Dazzle™ 594 anti-mouse Catalog No. 100246) for T-cells and F-480 antibody (APC/Cy7 anti-mouse Catalog o. 123117) for macrophages. All antibodies were used at 1:100 dilution and samples were stained for 30 mins. Analysis was done using FlowJo software (version 10). All subpopulations were analyzed independently under total live cells unless otherwise noted.

### Cell culture

Cell lines derived from the mutant Kras/p53 mouse model and human lung cancer cells H157 were cultured in RPMI1640 with 10% fetal bovine serum (FBS). Dasatinib and AZD0530 were a kind gift from Dr. Faye Johnson (MD Anderson). TGF-β was purchased from Cell Signaling (#8915LF). All blocking antibodies and Ig controls were purchased from BD Pharmingen and used at a final concentration of 8 μg/ml: Itgβ1 (BD 555002).

### Migration and Invasion Assays

Cells were seeded at 5 × 10^4^ per well in serum-free media in a 24-well Transwell or Matrigel plate (BD Biosciences, pore size 8 μm). RPMI with 10% FBS was placed in the lower chamber as chemoattractant and cells were allowed to migrate for 6 (H157 cells) or 16 hrs (murine cells) at 37 °C, 5% CO2. The migrated/invaded cells were stained with 0.1% crystal violet, captured in five microscopic fields at 4x magnification per well and counted. The results are represented as mean ± standard deviation and student’s t-test was performed for statistical significance. The graphs in each figure represent one experiment. Each assay was performed in triplicate.

### RT-PCR and western blotting

EVTs were isolated from the gels using cold PBS-EDTA. For dissolving the Matrigel/Collagen layer, Collagenase treatment was performed after the initial wash. Each well was incubated with Collagenase Type I (1,000 U/ml, Calbiochem #234153) for 30 min at 37 °C. Total RNA was isolated by TRIzol (Thermo Fisher Scientific) according to manufacturer’s protocol. RT-PCR assays were performed using SYBR Green PCR Master Mix (Thermo Fisher Scientific) and normalized to the L32 gene. Cell lysates were prepared according to the RIPA buffer protocol (CS9806), separated by sodium dodecyl sulfate-polyacrylamide gel electrophoresis, transferred to nitrocellulose membranes and probed with antibodies indicated. Antibodies were obtained from the following companies: Zeb1(sc-25388), N-cadherin (BD610921), E-cadherin (BD610182), p-Src Y416 (CS2101), Src (CS2108), p-Cortactin Y421 anti-mouse (Invitrogen44854), p-Cortactin Y421 anti-human (NBP1-60806), Cortactin (MP05-180), p-FAK Y397 (Invitrogen44624G), p-FAK Y576/577 (CS3281), p-FAK Y861(Invitrogen44-626G), p-FAK Y925 (CS3284), FAK (InvitrogenAHO0502), p-Paxillin Y118 (Abcam4833), Paxillin (Abcam2264), Itgβ1 (CS4706), p-p130Cas Y410 (CS4011), p130Cas (MP06-500), CRKL (MP05-414).

### Immunohistochemical staining

Formalin-fixed and paraffin embedded tissue sections were stained with p-SrcY^418^ (MP569373) and anti-rabbit biotinylated antibody (DAKO #0353).

## Supplementary information

Supplementary figures and legends

## Data Availability

All data discussed in this report is available from the authors on request.
