# Supplementary material for: A novel ex vivo tumor system identifies Src-mediated invasion and metastasis in mesenchymal tumor cells in non-small cell lung cancer
Source: Sci Rep. 2019 Mar 20;9:4819. doi: 10.1038/s41598-019-41301-2 (PMC6427036; doi:10.1038/s41598-019-41301-2)
Supplement: Supplementary file 1 — Supplementary figures and legends [file 41598_2019_41301_MOESM1_ESM.pdf]

**Title**

A novel *ex vivo* tumor system identifies Src-mediated invasion and metastasis in mesenchymal tumor cells in non-small cell lung cancer.

**Authors and Affiliations**

Aparna Padhye[1,2], Christin Ungewiss[1], Jared J. Fradette[1], B. Leticia Rodriguez[1], Jacob Albritton[4], Jordan Miller[4], Don L. Gibbons[1,3,*]

1. Department of Thoracic/Head and Neck Medical Oncology, The University of Texas MD Anderson Cancer Center, Houston, TX, USA.
2. MD Anderson Cancer Center UTHealth Graduate School of Biomedical Sciences, Houston, TX, USA.
3. Department of Molecular and Cellular Oncology, The University of Texas MD Anderson Cancer Center, Houston, TX, USA.
4. Department of Bioengineering, Rice University, Houston, Texas, USA.

***Corresponding author:** Don L. Gibbons, Department of Thoracic/Head and Neck Medical Oncology, The University of Texas MD Anderson Cancer Center, 1515 Holcombe Boulevard, Unit 432, Houston, TX 77030, USA, Phone: 713-792-9536, Fax: 713-792-1220, Email: dlgibbon@mdanderson.org

**Supplementary figure legends**

**Figure S1.** (A) Gating scheme to show different TME cells within primary tumors and EVTs. (B) Flow cytometry analysis to compare T cell subpopulations (CD8+ and CD4+) within EVTs and primary syngeneic tumors. No significant difference observed in percentage of CD3+T-cells but EVTs had higher percentage of CD8+ and lower percentage of CD4+ T cells compared to primary tumors. Further classification into exhausted CD8 T cells and regulatory CD4 T cells did not show significant differences between EVTs and primary tumors.

**Figure S2.** (A) EVTs derived from orthotopic lung tumors are cultured in Matrigel for 7 days and sphere size is quantified during that time. H&E staining of the lungs to show tumors. (B) Lung-EVTs are treated with TGFβ (5ng/ml) and demonstrate an increase in size and invasiveness Scale bar μM. Quantitative RT–PCR of indicated markers, shown as fold change upon TGFβ treatment.

**Figure S3.** (A) Quantification of invasive structures in response to alterations of the matrix. For each condition, 250 structures were scored for invasiveness. (B) Quantitative RT–PCR of indicated EMT markers show no change in response to Collagen I in the matrix. (C) Western blot analysis of EMT markers. (D) Non-metastatic, epithelial 393P_EVTs cultured in Matrigel as control and further treated with TGFβ or cultured in varying Collagen I mixtures. Quantification shows percentage of structures invasive in response to alterations. Western blot analysis on EMT markers. (E) Lung-EVTs are cultured in Matrigel or Matrigel/Collagen I with different concentrations of Collagen I for 5 days. (F) 344SQ_EVTs cultured in Matrigel and Matrigel/Collagen I (3 mg/ml) and treated with an ITGβ1-blocking antibody or IgM control for 5 days.

**Figure S4.** (A) Western blot analysis to show the EMT markers in the murine KP cell line panel (B) Western Blot analysis shows increased Src pathway activation in cell lines stably expressing Zeb1 which is inhibited upon treatment with dasatinib (50 nM). (C) Western Blot analysis of H157 cells treated for 24 hrs with dasatinib at 25 and 50 nM. (D) *In vitro* migration (black bar) and invasion (grey bar) assay of H157 cells treated with dasatinib (50 nM). (E) H157 cells grown in Matrigel/Collagen I and treated with dasatinib starting at day 0. Images taken at day 9.

**Figure S5.** (A) 344SQ_EVTs culture in Matrigel for 8 days. Images were taken at day 8. AZD0530 (3 μM) was added at the time of seeding and TGFβ (5 ng/ml) at day 4. (B) Size of EVTs and invasiveness of EVTs on TGFβ and AZD0530 treatments. (C) Western blot analysis of EVTs treated with AZD0530 and TGFβ at indicate concentrations.

# Figure S1

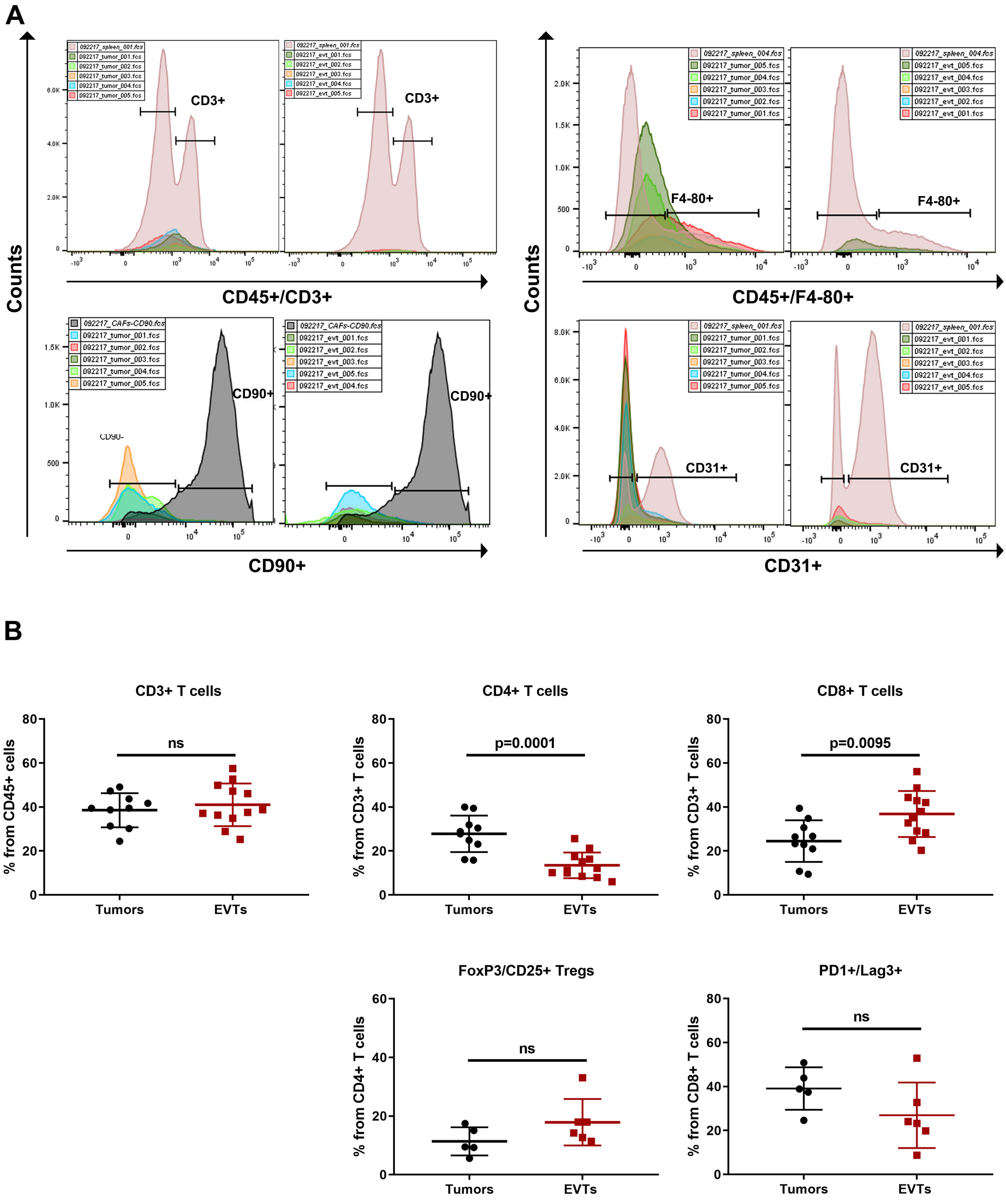

A
Counts
CD3+
CD45+/CD3+
CD90+
CD90-
CD90+
Counts
F4-80+
CD45+/F4-80+
CD31+
CD31+
B
CD3+ T cells
% from CD45+ cells
ns
Tumors
EVTs
CD4+ T cells
% from CD3+ T cells
p=0.0001
Tumors
EVTs
CD8+ T cells
% from CD3+ T cells
p=0.0095
Tumors
EVTs
FoxP3/CD25+ Tregs
% from CD4+ T cells
ns
Tumors
EVTs
PD1+/Lag3+
% from CD8+ T cells
ns
Tumors
EVTs

# Figure S2

A

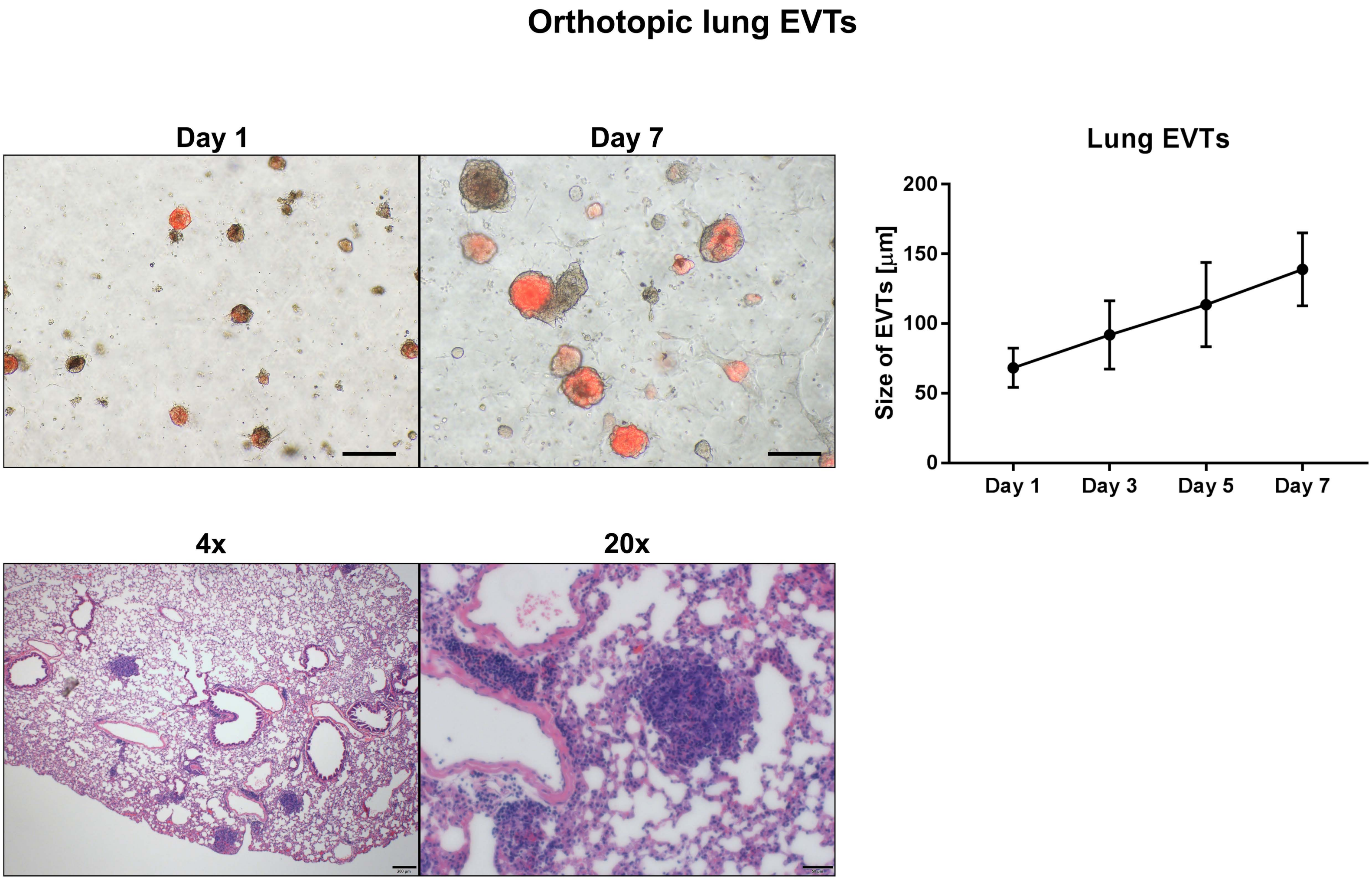

Orthotopic lung EVTs
Day 1
Day 7
Lung EVTs
200
150
100
50
0
Size of EVTs [μm]
Day 1
Day 3
Day 5
Day 7
4x
20x


B

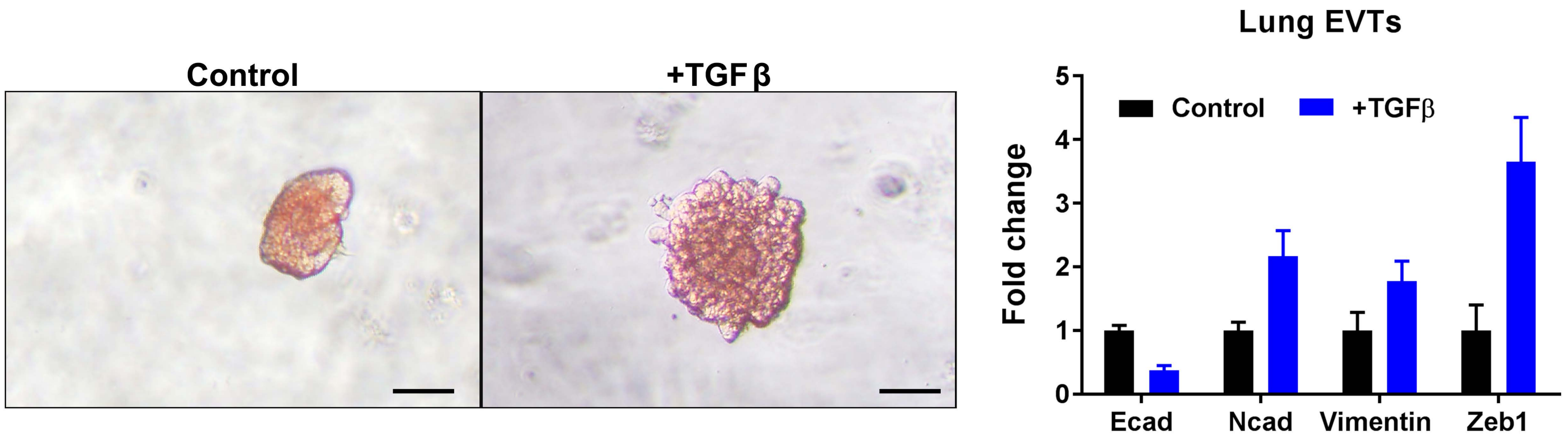

Control
+TGFβ
Lung EVTs
5
4
3
2
1
0
Fold change
Control
+TGFβ
Ecad
Ncad
Vimentin
Zeb1

# Figure S3

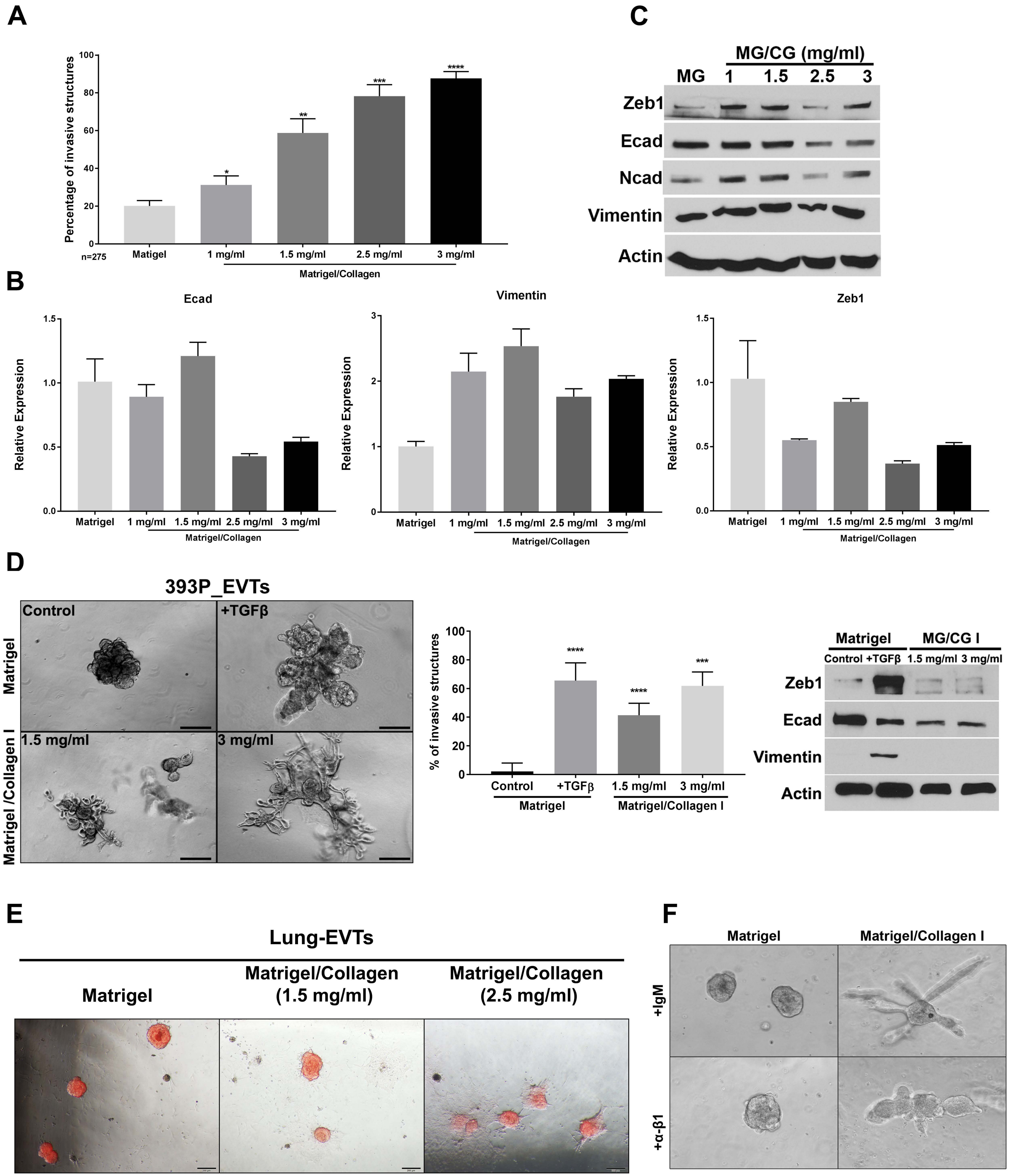
A
Percentage of invasive structures
100
80
60
40
20
0
*
**
***
****
n=275
Matigel
1 mg/ml
1.5 mg/ml
2.5 mg/ml
3 mg/ml
Matrigel/Collagen
C
MG/CG (mg/ml)
MG
1
1.5
2.5
3
Zeb1
Ecad
Ncad
Vimentin
Actin
B
Ecad
Vimentin
Zeb1
Relative Expression
1.5
1.0
0.5
0.0
3
2
1
0
Matrigel
1 mg/ml
1.5 mg/ml
2.5 mg/ml
3 mg/ml
Matrigel/Collagen
D
393P_EVTs
Control
+TGFβ
Matrigel
1.5 mg/ml
3 mg/ml
Matrigel /Collagen I
% of invasive structures
100
80
60
40
20
0
****
****
***
Control
+TGFβ
1.5 mg/ml
3 mg/ml
Matrigel
Matrigel/Collagen I
Matrigel
MG/CG I
Control
+TGFβ
1.5 mg/ml
3 mg/ml
Zeb1
Ecad
Vimentin
Actin
E
Lung-EVTs
Matrigel
Matrigel/Collagen
(1.5 mg/ml)
Matrigel/Collagen
(2.5 mg/ml)
F
Matrigel
Matrigel/Collagen I
+IgM
+α-β1

# Figure S4

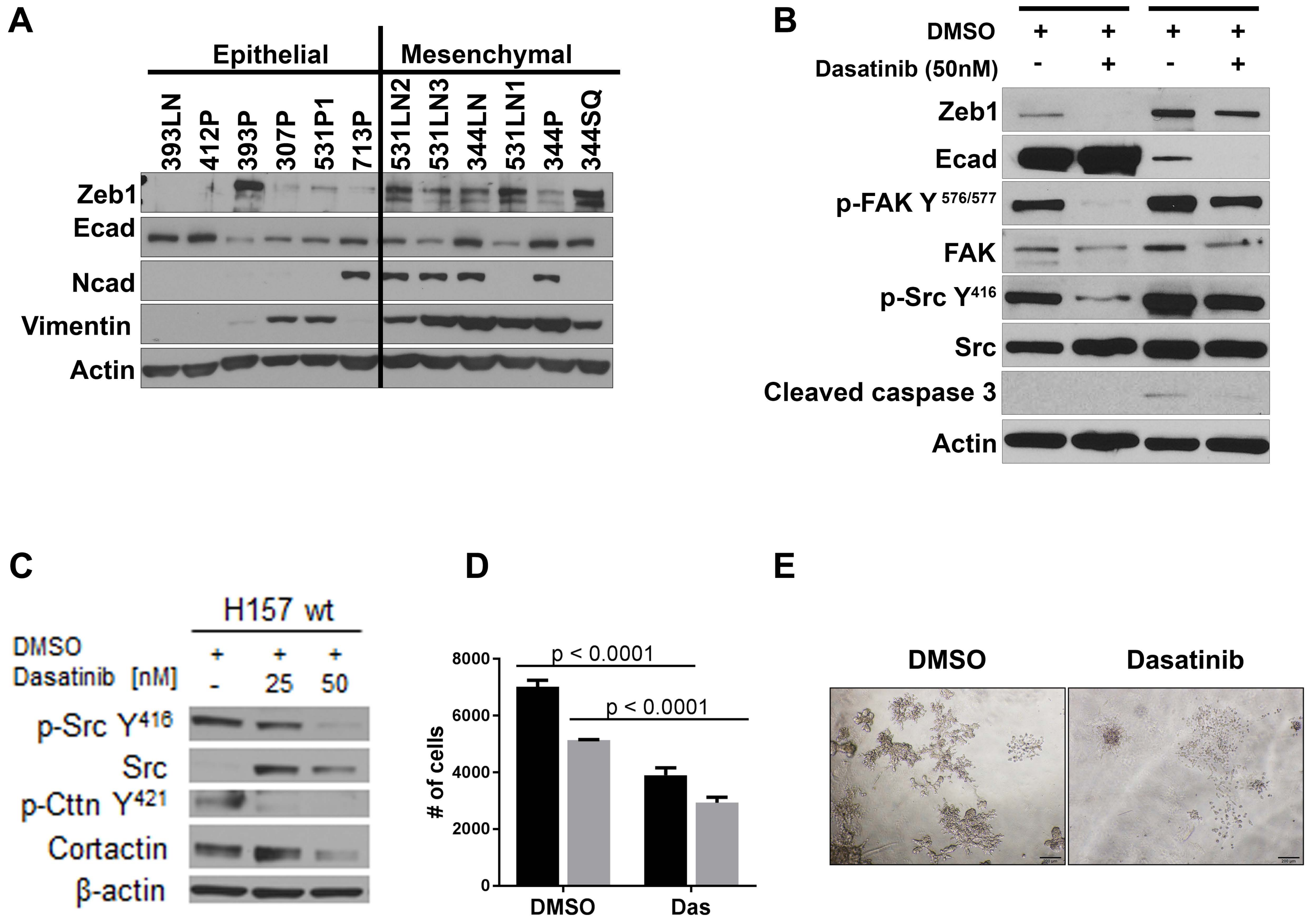
A
Epithelial
Mesenchymal
393LN
412P
393P
307P
531P1
713P
531LN2
531LN3
344LN
531LN1
344P
344SQ
Zeb1
Ecad
Ncad
Vimentin
Actin
B
393P_Vec
393P_Zeb1
DMSO + + + +
Dasatinib (50nM) - + - +
Zeb1
Ecad
p-FAK Y 576/577
FAK
p-Src Y416
Src
Cleaved caspase 3
Actin
C
H157 wt
DMSO + + +
Dasatinib [nM] - 25 50
p-Src Y416
Src
p-Cttn Y421
Cortactin
β-actin
D
p < 0.0001
p < 0.0001
# of cells
8000
6000
4000
2000
0
DMSO
Das
E
DMSO
Dasatinib

# Figure S5

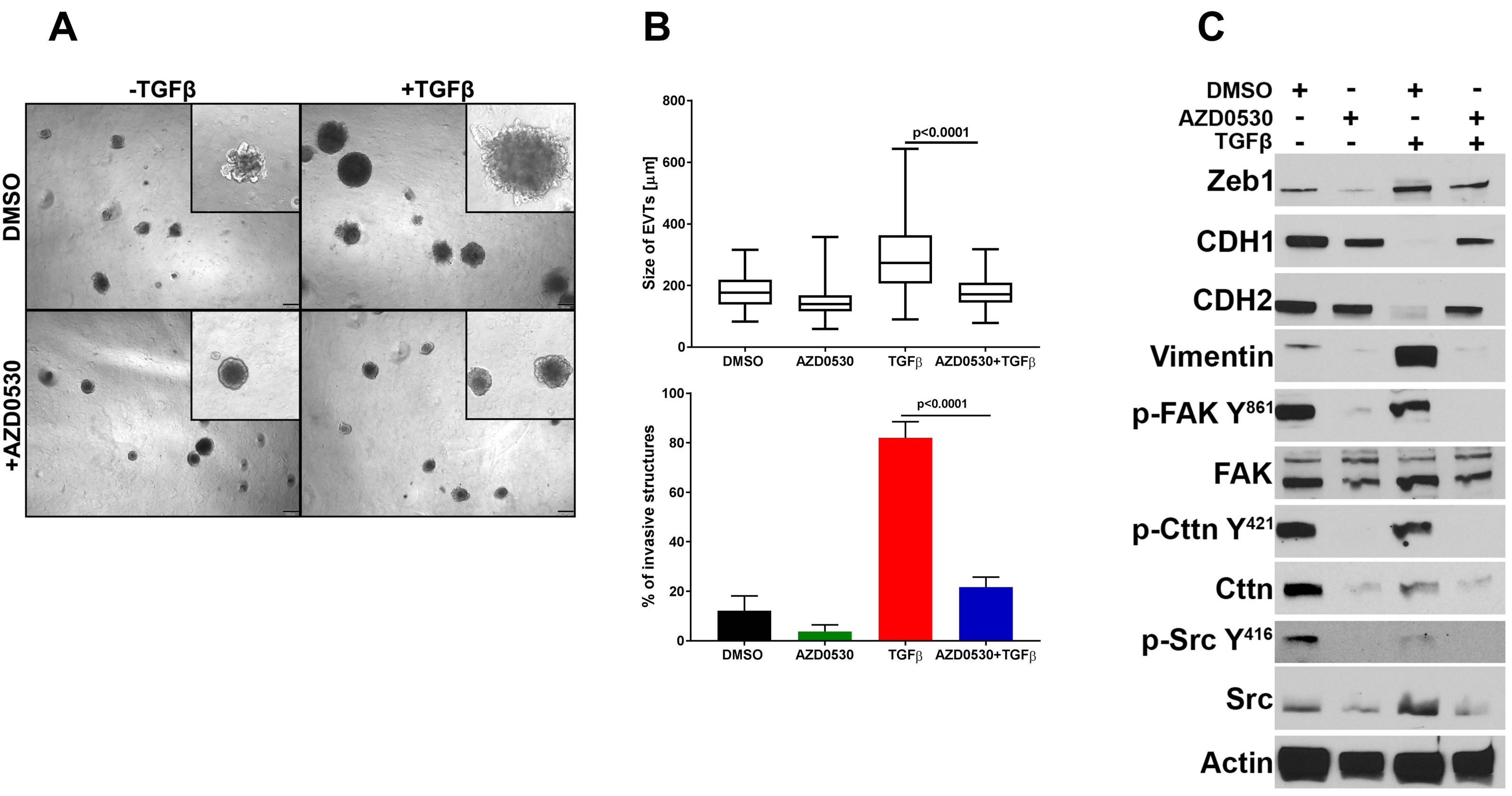
A
-TGFβ
+TGFβ
DMSO
+AZD0530
B
Size of EVTs [μm]
800
600
400
200
0
p<0.0001
DMSO
AZD0530
TGFβ
AZD0530+TGFβ
% of invasive structures
100
80
60
40
20
0
p<0.0001
DMSO
AZD0530
TGFβ
AZD0530+TGFβ
C
DMSO + - + -
AZD0530 - + - +
TGFβ - - + +
Zeb1
CDH1
CDH2
Vimentin
p-FAK Y861
FAK
p-Cttn Y421
Cttn
p-Src Y416
Src
Actin
